# The Role of DNA Double-Strand Breaks in Spontaneous Homologous Recombination in S. cerevisiae

**DOI:** 10.1371/journal.pgen.0020194

**Published:** 2006-11-10

**Authors:** Gaëlle Lettier, Qi Feng, Adriana Antúnez de Mayolo, Naz Erdeniz, Robert J. D Reid, Michael Lisby, Uffe H Mortensen, Rodney Rothstein

**Affiliations:** 1 Center for Microbial Biotechnology, BioCentrum-DTU, Technical University of Denmark, Lyngby, Denmark; 2 Department of Genetics and Development, Columbia University Medical Center, New York, New York, United States of America; 3 Department of Genetics, Institute of Molecular Biology and Physiology, University of Copenhagen, Copenhagen, Denmark; Brandeis University, United States of America

## Abstract

Homologous recombination (HR) is a source of genomic instability and the loss of heterozygosity in mitotic cells. Since these events pose a severe health risk, it is important to understand the molecular events that cause spontaneous HR. In eukaryotes, high levels of HR are a normal feature of meiosis and result from the induction of a large number of DNA double-strand breaks (DSBs). By analogy, it is generally believed that the rare spontaneous mitotic HR events are due to repair of DNA DSBs that accidentally occur during mitotic growth. Here we provide the first direct evidence that most spontaneous mitotic HR in Saccharomyces cerevisiae is initiated by DNA lesions other than DSBs. Specifically, we describe a class of *rad52* mutants that are fully proficient in inter- and intra-chromosomal mitotic HR, yet at the same time fail to repair DNA DSBs. The conclusions are drawn from genetic analyses, evaluation of the consequences of DSB repair failure at the DNA level, and examination of the cellular re-localization of Rad51 and mutant Rad52 proteins after introduction of specific DSBs. In further support of our conclusions, we show that, as in wild-type strains, UV-irradiation induces HR in these *rad52* mutants, supporting the view that DNA nicks and single-stranded gaps, rather than DSBs, are major sources of spontaneous HR in mitotic yeast cells.

## Synopsis

The genome of any organism is constantly damaged as an inevitable result of its own metabolism and exposure to irradiation. For that reason all organisms have developed many DNA repair systems to cope with the different types of DNA damage that challenge the stability of their genomes during daily life. One of these repair mechanisms is based on homologous recombination (HR), which, as a side effect, may result in loss of heterozygosity. For example, if a diploid organism harbors one functional and one dysfunctional copy of an important gene, DNA repair by HR may lead to a cell where both copies are defective. Since loss of heterozygosity plays a major role in tumorigenesis in higher eukaryotes, it is important to understand what types of DNA damage trigger HR most efficiently. In this paper, the authors have used a yeast-based system to investigate this topic, and based on mutations that separate the functions of Rad52 (a protein that is essential for HR) they conclude that DNA double-strand breaks are not the lesions that initiate most HR, but rather it is due to DNA nicks and single-stranded DNA regions.

## Introduction

Spontaneous mitotic homologous recombination (HR) plays an important role in securing the integrity of the genome [1–3]. On the other hand, this process may lead to loss of heterozygosity, which plays a major role in tumorigenesis in higher eukaryotes. For these reasons, it is important to understand how spontaneous mitotic HR is initiated. Much of our understanding of how HR is initiated comes from meiotic studies. Here, the high level of HR is due to the programmed formation of a large number of DNA double-strand breaks (DSBs) [4]. These breaks are produced in early prophase by the coordinated action of a number of proteins including Spo11, which is believed to be directly responsible for strand cleavage [5–7]. Likewise, DSBs have been shown to promote HR in mitotic cells. For example, the well-characterized ability of haploid Saccharomyces cerevisiae cells to switch mating type is the result of an HR event that is initiated by a DSB produced by the HO-endonuclease [8]. More generally, γ-irradiation, which induces DSBs in the genome, increases the frequency of HR [3], and linear DNA molecules transformed into a cell may integrate into the genome via HR [9,10]. Importantly, both DSB repair and spontaneous HR are dependent on the activities encoded by the genes in the *RAD52* epistasis group [3], which strongly indicates that the two processes have a common biochemistry. It has therefore generally been assumed that the source of spontaneous HR is a DSB that occurs accidentally during the cell cycle. However, this assumption remains speculative as spontaneous HR is rare and the triggering lesion has never been identified [1]. It is known that repair of other types of lesions may result in HR. For example, HR is stimulated by UV-irradiation that mostly produces pyrimidine dimers [11,12]. Moreover, in Escherichia coli it has been demonstrated that single-stranded gaps are potent substrates for HR [13–16]. Although many different lesions have been shown to trigger HR, the nature of the molecular event(s) that causes most spontaneous mitotic HR remains unclear.

Rad52 is important for DSB repair and all types of HR in *S. cerevisiae,* and to understand the role of Rad52 in these processes we have previously performed a comprehensive alanine scan mutation study. This plasmid-based screen identified a class of nine *rad52* mutants (class C mutants) that are sensitive to γ-irradiation, yet maintain wild-type levels of mitotic HR [17]. This result suggests that the role of Rad52 in DSB repair and spontaneous HR can be separated. To investigate this possibility, we have introduced each of the nine separation-of-function *rad52* mutations into the genome of S. cerevisiae for further characterization. By analyzing the repair of different types of defined DSBs we have shown that *rad52* class C mutants indeed are defective in DSB repair and that repair is blocked at a stage after the recruitment of both Rad51 and mutant Rad52 to the break. In contrast, all class C mutants perform mitotic HR at wild-type or higher levels and this activity is independent of the presence of Rad59, a Rad52 paralog in S. cerevisiae. Interestingly, mitotic HR is efficiently induced by UV-irradiation in the *rad52* class C mutant strains. Together our results are consistent with a view that DNA nicks and single-stranded gaps, rather than DSBs, are major sources of spontaneous HR in mitotic yeast cells.

## Results

### Initial Characterization of *rad52* Separation-of-Function Mutants

**Figure 1:**
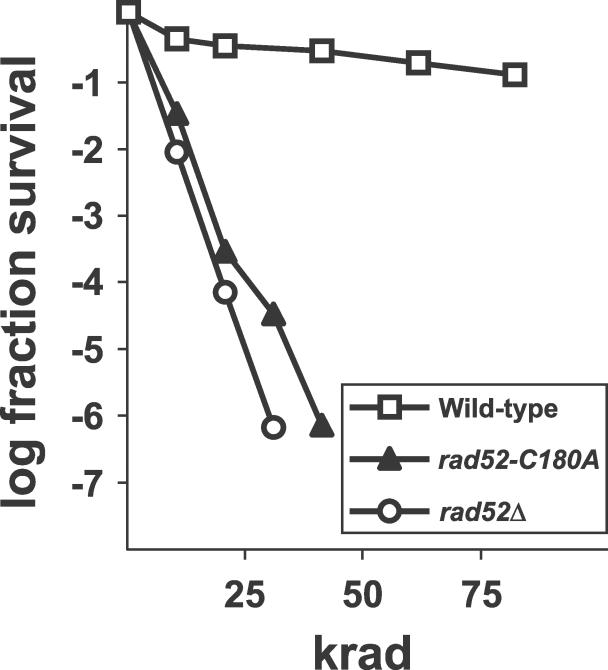
γ-Ray Sensitivity of *rad52-C180A, rad52Δ,* and Wild-Type Strains

Initially, we addressed the possibility that the *rad52* separation-of-function phenotype identified in our previous screen [17] was caused by the fact that all mutant Rad52 species were ectopically expressed from a plasmid. Therefore, we replaced the endogenous *RAD52* gene by each of the nine *rad52* class C alleles and the resulting strains were individually tested for their ability to repair γ-ray-induced DNA damage and to perform mitotic heteroallelic HR. First, we confirmed that all *rad52* class C mutant strains are γ-ray sensitive (Figure 1 and Table 1). In fact, most *rad52* class C mutant strains display sensitivities comparable to that measured for *rad52*Δ strains. Next, we confirmed that spontaneous HR occurs at a high rate in all *rad52* class C mutant strains. Hence, all diploid *rad52* class C mutant strains display high interchromosomal-heteroallelic HR rates (Table 2). In fact, four strains, *rad52-Y66A, -R70A, -W84A,* and -R*156A* are significantly hyper-recombinogenic (3- to 4-fold). Moreover, we measured the rate of HR between two directly repeated *leu2* heteroalleles, *leu2-*Δ*Eco*RI and *leu2-*Δ*Bst*EII in haploid strains (Table 2). In this assay, two *rad52* class C mutant strains, *rad52-R85A* and *-R156A,* produced HR rates identical to that obtained with wild-type strains. The remaining *rad52* class C mutant strains were slightly hypo-recombinogenic and the largest decrease, 3-fold, was observed for *rad52-C180A.* Importantly, even with *rad52-C180A* strains, the direct-repeat HR rate is 10-fold higher than that observed for *rad52*Δ strains. Taken together we have confirmed the separation of Rad52 functions in HR and γ-ray damage repair in strains where the *rad52* class C mutations are integrated at the *RAD52* locus.

**Table 1:**
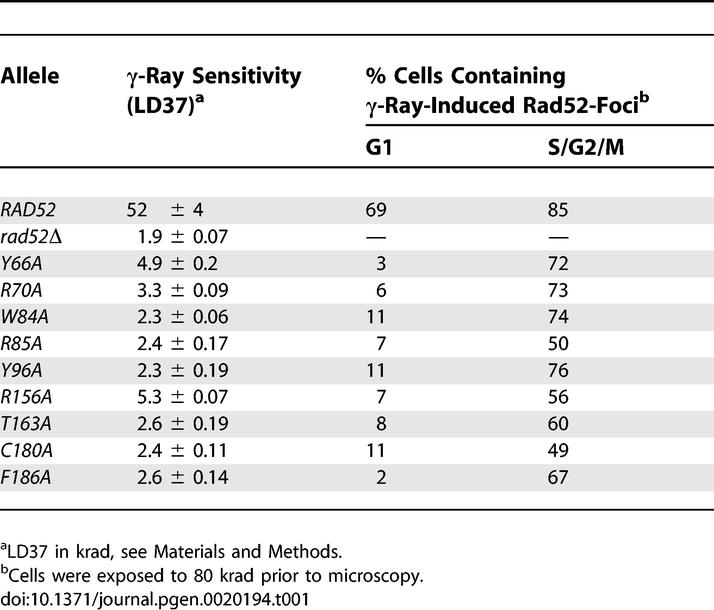
Effect of γ-Irradiation on *rad52* Mutant Strains

**Table 2:**
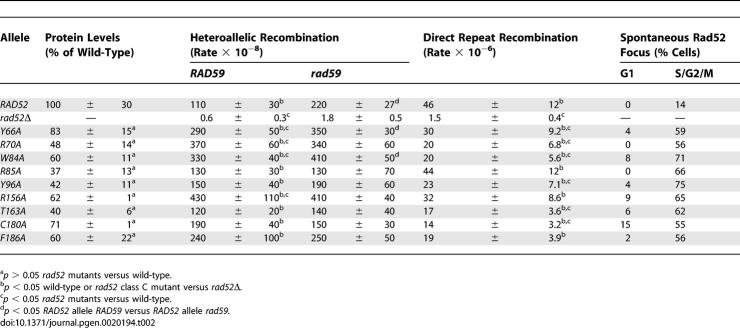
Effects of *rad52* Mutations on Rad52 Protein Levels, Inter- and Intrachromosomal Recombination, and Rad52 Focus Formation in Mitotic Cells

### The *rad52* Class C Mutant Phenotype Is Not Caused by Reduced Rad52 Protein Levels

The separation-of-function phenotype of *rad52* class C mutants could potentially be related to instability of the mutant proteins. For example, it could be argued that repair of γ-ray-induced damage requires more Rad52 protein compared to the amount required to maintain rare mitotic HR events. However, protein blot analysis showed that the Rad52 levels are slightly reduced, but none of these reductions are statistically different from the protein level found for wild-type strains (Table 2). Moreover, we have previously shown that γ-ray survival is reduced only 2- to 3-fold and HR 4-fold in strains, which produce 25% of the wild-type level of Rad52 protein [18]. Thus, the separation-of-function phenotype of *rad52* class C mutations cannot be explained by slightly lowered Rad52 protein concentrations.

### Mitotic Recombination in *rad52* Class C Mutants Does Not Depend on Rad59

S. cerevisiae contains a truncated Rad52 paralog, Rad59. A mutation in *RAD52 (rad52-R70K)* has previously been described to cause a synthetic defect with *rad59*Δ suggesting that the two proteins have overlapping functions [19,20]. All of the *rad52* class C mutations are located in a region of Rad52 that is homologous to Rad59. Thus, we investigated whether the ability of the *rad52* class C mutants to perform HR was due to the ability of Rad59 to substitute for an impaired Rad52 function. However, the rates of interchromosomal heteroallelic HR obtained for wild-type and all the *rad52* class C mutant strains in the absence of Rad59 are mostly indistinguishable from the rates obtained in its presence (Table 2). In fact, three strains, *RAD52, rad52-Y66A,* and *rad52-W84A* show small, 1.5- to 2-fold, but significant, increases of the HR rate in the absence of Rad59.

### *rad52* Class C Mutant Strains Fail to Repair Defined DNA DSBs

**Figure 2:**
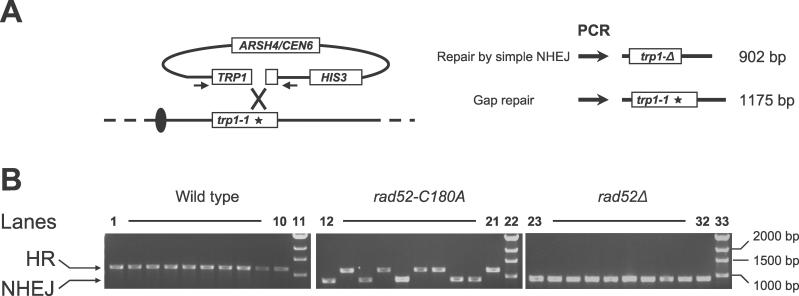
Gap Repair Is Impaired in *rad52* Class C Mutant Strains (A) Graphical representation of the assay used to address the nature of gap closure events. Gapped pRS413-TRP1 repaired by HR. This event results in transfer of the *trp1–1* mutation in the genome to the plasmid. The position of the *trp1–1* mutation relative to the gapped *TRP1* plasmid-borne sequence is indicated by an asterisk. Repair by simple NHEJ (without any further rearrangement/deletion of plasmid DNA) results in a 273-bp deletion in *TRP1*. The two types of events were distinguished by PCR using a plasmid specific primer pair, indicated as small arrows. The PCR product sizes expected from transformants resulting from a gapped plasmid that has been repaired by HR and from one that has been closed by NHEJ are shown. (B) Gel electrophoresis analysis of PCR fragments obtained from strains transformed with gapped pRS413-TRP1. Arrows point to the band sizes expected if the gapped plasmid has been repaired by HR or by NHEJ. Representative analyses of ten transformants obtained with wild-type strains (lanes 1–10), ten with *rad52-C180A* strains (lanes 12–21), and ten with *rad52*Δ strains (lanes 23–32). Sizes of relevant bands in the DNA marker (lane 11, 22, and 33) are indicated.

It is generally assumed that the lethality of *rad52Δ* strains after exposure to γ-rays is the consequence of unrepaired DSBs. However, besides DSBs, γ-irradiation causes a variety of DNA modifications such as clustered base-modifications, abasic sites, and nicks, as well as DNA-DNA and DNA-protein crosslinks [21–24]. Repair of such lesions may rely on Rad52 functions that differ from those required for efficient DSB repair. It is therefore possible that *rad52* class C mutant strains are capable of repairing DSBs, but die after γ-irradiation because of their inability to repair other types of lesions. Assuming that a DSB is required to initiate HR, this scenario would explain why *rad52* class C mutants can be γ-ray sensitive yet at the same time be HR-proficient. To explore this possibility, *rad52* class C mutant strains were investigated for their ability to repair and survive a single well-defined DSB. First, the ability of *rad52* class C mutants to catalyze plasmid gap repair was determined (Figure 2A). In wild-type strains, all transformants tested (20 out of 20, see Table 3) contained a plasmid that was sealed by HR using the genomic *trp1–1* allele as template (ten are shown in Figure 2B). In contrast, with *rad52* class C and *rad52*Δ mutant strains, the repair frequencies are reduced 10–100- and 250-fold, respectively, compared to the efficiency obtained with wild-type strains, indicating that plasmid re-circularization occurs inefficiently in these strains. The low repair frequencies in *rad52* class C and *rad52*Δ mutants are likely due to their inability to close a gapped plasmid by HR. Indeed, with the exception of *rad52-R70A,* the majority of the transformants tested contained plasmids that were generated by non-homologous end-joining (NHEJ) rather than by HR (Table 3). In addition, we obtained a few aberrant transformants with *rad52*Δ and *rad52* class C mutant strains that were not formed as the result of plasmid repair by simple NHEJ or plasmid-gap repair (Table 3). For these events, the PCR analysis produced either a band of a size that is significantly different from that expected if the two plasmid ends simply joined; hence, indicating a larger deletion/rearrangement event had taken place, or no PCR fragment could be recovered. For one mutant strain, *rad52-Y96A,* this class constitutes the major type of events.

**Table 3:**
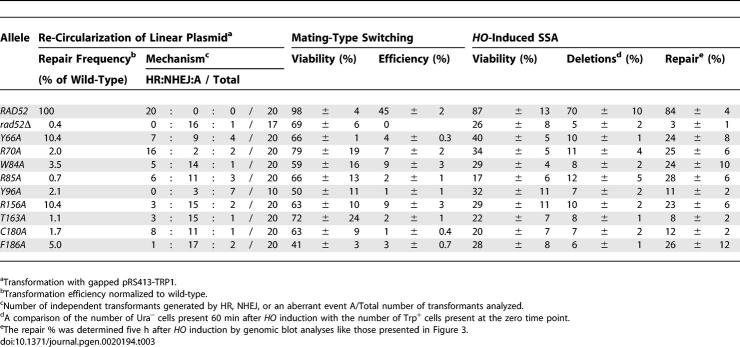
Effects of *rad52* Mutations on the Repair Efficiency of Defined DNA DSBs

**Figure 3:**
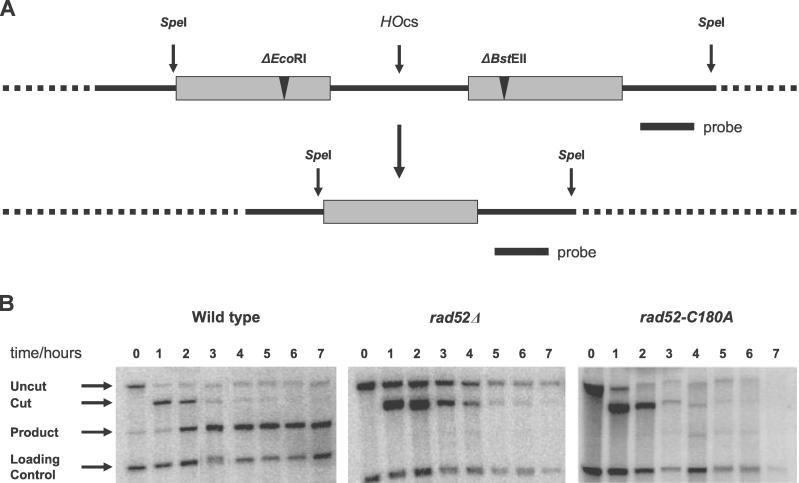
Kinetics of the Repair of a DSB Produced between Directly Repeated *leu2* Heteroalleles (A) Graphical representation of the assay used to follow HO-endonuclease-induced direct-repeat HR. The positions of the *leu2-*Δ*Bst*EII and *leu2-*Δ*Eco*RI heteroalleles are indicated by black wedges. The position of the *HO* cut-site, *HO*cs, is indicated by an arrow. The product resulting from DSB repair by SSA is shown below the direct-repeat assay. Note that the product is given as a wild-type sequence, but it could also contain any combination of the *leu2-*Δ*Bst*EII and *leu2-*Δ*Eco*RI alleles, as the analysis performed here does not discriminate between these possibilities. Arrows labeled *Spe*I indicate the positions of the *Spe*I cut-sites used to release the region from its chromosomal context for genomic blot analysis. Horizontal bars represent the location of the probe used for genomic blot analysis. (B) The DSB was produced by induction of the HO-endonuclease and repair was analyzed in three different strain backgrounds, wild-type, *rad52*Δ*,* and *rad52-C180A,* as indicated. In each strain, the kinetics of three DNA species in the process was followed: uncut DNA, cut DNA, and product. The positions of these species are indicated by arrows. A DNA fragment serving as loading control (see Materials and Methods) is also visualized. The number above each lane indicates the time point after induction of the HO-endonuclease in hours.

We next investigated the ability of the *rad52* class C mutants to repair a single chromosomal DSB induced by the HO-endonuclease at the mating-type locus. To survive, a cell must repair the break by gene conversion using either *HMLα* or *HMR* **a** as template [25]. We measured the ability of all *rad52* class C mutants to survive and switch mating type after transient induction of the HO-endonuclease (Table 3). After this treatment, essentially all wild-type cells survive and approximately half of them switch mating type. In contrast, significant amounts of all *rad52*Δ and *rad52* class C mutant cells (20%–40%) die after HO-endonuclease induction indicating that they suffered a DNA DSB, which failed to be repaired. The surviving cells likely failed to produce a DSB at the *MAT* locus after induction as none of the *rad52*Δ survivors and only few 1% *(rad52-Y96A* and *rad52-C180A)* to 9% *(rad52-W84A)* of the *rad52* class C mutant survivors switched mating type. Thus, even a single DSB is inefficiently repaired in *rad52* class C mutant strains. We also compared the ability of wild-type, *rad52*Δ*,* and one of the *rad52* class C mutant strains, *rad52-C180A,* to survive sustained expression of the HO-endonuclease in a spot assay. For wild-type strains, continuous expression of the HO-endonuclease caused a 10-fold reduction in viability. In contrast, the viability of *rad52*-C180A and *rad52*Δ strains was reduced an additional three orders of magnitude (Figure S1).

We also investigated the ability of *rad52* class C mutants to repair a DSB induced by the HO-endonuclease between two directly repeated sequences (Figure 3A). In wild-type strains, such a DSB is preferentially and efficiently repaired via the single-strand-annealing (SSA) pathway where the break is sealed at the expense of the intervening sequence [26]. In agreement with this, most of the wild-type cells survive induction of the HO-endonuclease and 70% of the survivors lose the sequence between the two repeats indicating successful repair by SSA (Table 3). In contrast, the viability of all *rad52* class C mutant strains was reduced to a level similar to that obtained for *rad52*Δ strains (Table 3) and among the survivors only a few of the cells contained a deletion event (6%–12%). This result suggests that *rad52* class C mutants are defective in SSA. To investigate this possibility in more detail, the fate of the HO-induced break was determined at the DNA level for all *rad52* class C mutant strains. Specifically, genomic blot analysis was used to measure three different DNA species: intact DNA, cut DNA, and the repair product. Consistent with an earlier study [27], we observed that the cut DNA is efficiently repaired in more than 80% of the wild-type cells within 5 h. In contrast, *rad52*Δ strains repaired less than 3% of the cleaved DNA in a similar time span. This explains the high lethality in the absence of Rad52. By performing the same analysis for the *rad52* class C mutant strains, we find that they all fail to produce wild-type levels of repaired DNA (Figure 3B and Table 3). In strains *rad52*-*Y66A,* -*R70A,* -*W84A,* -*R85A,* -*R156A,* and -*F186A* approximately 25% of the breaks are sealed to form a product, whereas in *rad52-Y96A, -T163A,* and *-C180A* approximately 10% of the breaks are repaired. Accordingly, most of the *rad52* class C mutant cells die after induction of the break, simply because they fail to join the resulting two ends. In addition, as is the case with *rad52*Δ strains, we note that cut DNA disappears as a function of time in *rad52* class C mutant strains despite the fact that no corresponding amount of product is being formed (Figure 3 and unpublished data). In *rad52*Δ strains, this phenomenon is due to the continuous degradation of the 5′-strand at the breaks. Eventually the single-stranded region expands to include the restriction enzyme, *Spe*I, cut site, which is used to liberate the detectable fragment for the genomic blot analysis. Accordingly, this fragment is shifted in the gel [26–28].

### *rad52* Class C Mutant Strains Are Sensitive to Camptothecin

**Figure 4:**
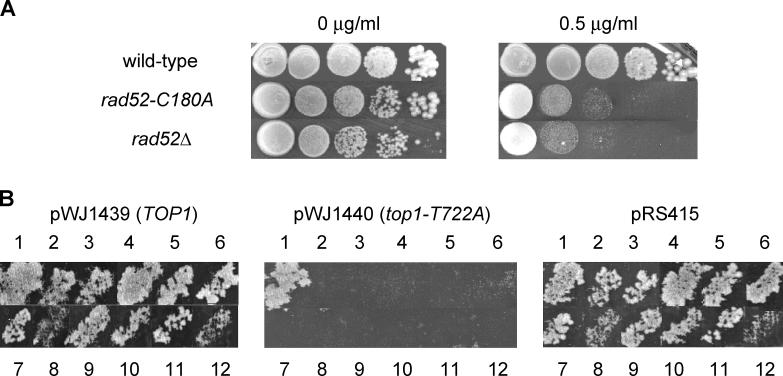
*rad52* Class C Mutant Strains Are Sensitive to Stable Topoisomerase-Induced DNA Nicks (A) Serial 10-fold dilutions of wild-type, *rad52-C180A,* and *rad52* null strains were spotted on solid medium containing no camptothecin or 0.5 μg/ml camptothecin as indicated. (B) pWJ1439 *(TOP1),* pWJ1440 *(top1-T722A),* and pRS415 were transferred to wild-type, *rad52Δ,* and all *rad52* class C mutant strains by plasmoduction as described in Materials and Methods. The positions of wild-type, *rad52*Δ*, rad52-Y66A, -R70A, -W84A, -R85A, -Y96A, -R156A, -T163A, -C180A,* and *-F186A* strains on selective plates are indicated by numbers: 1, (2 and 3), 4, 5, 6, 7, 8, 9, 10, 11, and 12, respectively.

A lesion similar to a DSB may also be formed if a cell replicates a nicked template since a free DNA end is formed when a replication fork collapses at a nick. The replication fork can be restored if the free DNA end invades a homologous sequence in a process that involves recombination. Unlike repair of a DSB, this event only involves single-end invasion [29,30]. To test whether this type of DNA lesion could account for the HR observed in *rad52* class C mutant strains, we determined their ability to survive exposure to the anti-tumor drug camptothecin. This drug stabilizes the covalent DNA-Top1 intermediate that forms during the catalytic DNA nicking-closing cycle of Top1 [31,32]. Accordingly, addition of camptothecin leads to the formation of stable nicks in the genome that may be converted into recombinogenic DNA ends when the genome is replicated [33]. At 0.5 μg/ml camptothecin, the viability of *rad52* class C and *rad52*Δ mutant strains is reduced 10- to 1,000-fold and 1,000-fold, respectively, compared to wild-type strains (Figure 4A, and unpublished data). Similar stable DNA nicks are generated in strains expressing *top1-T722A* since the nicking-closing equilibrium of this mutant is shifted towards nicking [34]. To substantiate the results obtained by exposing *rad52* class C mutants to camptothecin, we tested the ability of *rad52* class C mutant strains to survive expression of the *top1-T722A* mutant (Figure 4B). Plasmid-borne copies of *TOP1, top1-T722A,* or a vector control were transferred into haploid *RAD52, rad52*Δ, and *rad52* class C mutant strains by plasmoduction (see Materials and Methods). All strains tested were competent for plasmoduction as they took up the vector control and *TOP1* expression plasmids as indicated by growth on the selective media (Figure 4B). Consistent with previous results, the *top1-T722A*-containing plasmid could be transferred to wild-type strains, but not to *rad52*Δ strains [35], showing that *rad52*Δ strains do not survive DNA damage created by expression of the *top1-T722A* allele. As with *rad52*Δ strains, the *top1-T722A* plasmid failed to transfer to the *rad52* class C mutant strains as indicated by lack of growth on the selection plates (Figure 4B). Taken together, these data suggest that *rad52* class C mutant strains are deficient in repair of single-ended DSBs generated by a progressing replication fork.

### *rad52* Class C Mutant Strains Perform Break-Induced Replication with Reduced Efficiency

We also investigated the possibility that a selected *rad52* class C mutant, *rad52-C180A,* could rescue a linearized plasmid by a reaction that involves break-induced replication (BIR), by using a chromosome fragmentation assay. In this assay, one end of the plasmid contains TG_1–3_ repeats, which will be rescued by de novo telomere addition, the other end is rescued in a BIR event that involves one-ended invasion at the D8B region of Chromosome III [36,37]. Davis and Symington found that the efficiency of BIR in this assay is reduced at least 4,000-fold in *rad52Δ* strains, and in agreement with this we did not recover any transformants that result from BIR in the absence of Rad52. On the other hand, with *rad52-C180A* strains, we find that the BIR efficiency is only 2.7-fold reduced compared to wild-type indicating that the efficiency of a one-ended invasion is only mildly affected in class C mutants.

### Rad52 Class C Mutant Protein Forms Spontaneous Foci during the Mitotic Cell Cycle

**Figure 5:**
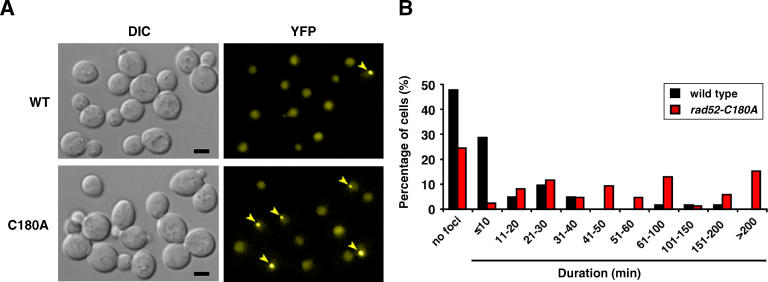
Duration of Spontaneous Rad52-YFP and Rad52-C180A-YFP Foci (A) Rad52-YFP and Rad52-C180A-YFP foci are formed in small-budded cells in mitotically growing cultures. Arrowheads point to Rad52-YFP foci. Scale bar, 3 μm. (B) The percentage of cells that do not develop a Rad52 focus during a cell cycle is shown in the left side of the histogram. The percentage of cells that do form a Rad52 focus is shown in the right side of the histogram as a distribution arranged according to the duration of the Rad52 focus observed in individual cells. Each column represents the percentage of cells that have turned the Rad52 focus over in the time frame indicated. Results from *RAD52* and *rad52-C180A* strains are shown as indicated. Median duration of Rad52 foci is 8 min for the wild-type and 57 min for *rad52-C180A.*

We have previously used a biologically functional Rad52-YFP (yellow fluorescent protein) fusion protein to monitor DNA repair in individual wild-type cells. This is possible as Rad52-YFP accumulates to form a bright focus at a lesion during S/G2/M-phase [38,39]. Like wild-type strains, a subset of S/G2/M-phase cells expressing class C mutant Rad52-YFP contains a spontaneous focus. This is consistent with *rad52* class C mutants being proficient for spontaneous HR (Table 2 and Figure 5A). In fact, the number of cells containing a repair focus is typically 5- to 6-fold higher than for wild-type strains. This phenomenon could be due to either more cells on average forming foci or to foci lasting longer in *rad52* class C mutant strains. To distinguish between these possibilities, *rad52-C180A-YFP* was analyzed in more detail by time-lapse microscopy (Figure 5B). The results from this analysis suggest that repair in the *rad52-C180A-YFP* mutant strain is slower than in wild-type strains. Specifically, 50% of all repair foci are processed within 10 min in wild-type cells and 90% within 1 h. In comparison, *rad52-C180A-YFP* cells need approximately 1 and 6 h to process 50% and 90% of all repair foci, respectively. The longer duration of Rad52-C180A-YFP foci likely reflects slow repair. In most cells, Rad52-C180A-YFP foci eventually disappear and cell division proceeds as normal indicating that the spontaneous Rad52-C180A-CFP foci represent active repair of spontaneous DNA lesions and not inactive Rad52 aggregates. Overall, the percentage of cells forming foci per cell cycle is higher in *rad52-C180A* strains (76%) compared to wild-type strains (53%) indicating that the number of lesions is slightly increased in *rad52-C180A* strains or that some foci in wild-type cells are too short-lived to be registered in the analysis. Finally, unlike wild-type strains most of the *rad52* class C mutant strains (seven out of nine) contain Rad52-YFP foci at a low frequency in G1 cells. Since yeast has been shown to adapt to the DNA damage checkpoint at G2/M [40,41] such foci are likely the result of long-lasting Rad52 foci being transmitted into G1 cells after adaptation to the G2/M checkpoint.

### A Rad52 Class C Mutant Protein and Rad51 Co-Localize at a Defined DNA DSB

**Figure 6:**
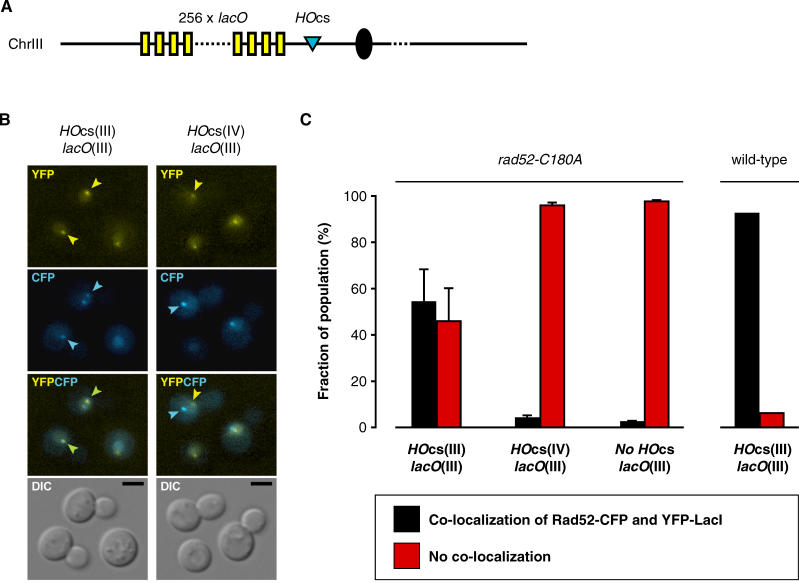
Rad52-C180A-CFP Is Recruited to a Specific DNA Double-Strand Break (A) Assay in Chromosome III for visualizing an HO-endonuclease inducible DSB. Yellow boxes: *lacO* sites. Cyan triangle: HO cut-site (HOcs). Solid circle: centromere. (B) Localization of a Rad52–CFP focus to an HO-endonuclease-induced DSB. The panels show YFP, CFP, CFP/YFP-merged, and DIC images of representative cells of a strain expressing Rad52-C180A-CFP in a strain with a *lacO* tandem array next to an HOcs on Chromosome III (left, strain W4021-20A) and in a strain with an HOcs on Chromosome III and a *lacO* tandem array on Chromosome IV (right, strain W4341-16A). The *lacO* tandem array is visualized by LacI-YFP as a yellow focus. The marked foci (arrowheads) in the left panels are examples of Rad52/LacI co-localization and the arrowheads in the example on the right indicate the absence of co-localization when the *lacO* elements and the HOcs are on different chromosomes. Scale bar, 3 μm. (C) Quantitative analysis of co-localization between Rad52-CFP and YFP-LacI foci. Chromosomal locations of the HOcs and the *lacO* tandem array are given below the histogram columns. As a control, strain W4341-6D with no HOcs was analyzed. The wild-type dataset shown for comparison is from [39].

The failure of *rad52* class C mutant strains to repair γ-ray-induced damage as well as defined DSBs could be explained if these lesions were never recognized by the mutant Rad52 species. To test this possibility, all class C *rad52-YFP* mutant strains were γ-irradiated with a dose of 80 krad to investigate whether this would induce class C Rad52-YFP focus formation (Table 1). As previously observed, un-irradiated wild-type G1 cells rarely, if ever, form repair foci. However, after irradiation, 69% of wild-type G1 cells display one or more bright foci [38]. This may represent an abnormal stress situation where the system that normally prevents Rad52-dependent DNA repair to occur in G1 is overwhelmed or bypassed. Interestingly, γ-irradiation did not increase the number of *rad52* class C mutant G1 cells containing a class C Rad52-YFP focus. In S/G2/M cells, which represent the population of cells where Rad52 dependent repair normally occurs, the number of wild-type cells that contain at least one Rad52-YFP focus increases 6-fold after γ-irradiation. In contrast, no significant increase is observed with *rad52* class C mutant cells (compare Tables 1 and 2). These results suggest that class C mutant Rad52-YFP proteins are not recruited to γ-ray-induced DSBs. However, this conclusion may be flawed because the majority of *rad52* class C mutant S/G2/M cells already contain a repair focus before irradiation making it hard to determine whether any additional foci were formed. We therefore decided to investigate whether a selected Rad52 class C mutant protein, Rad52-C180A-CFP (cyan fluorescent protein), is recruited to an HO-inducible cut-site. In strains expressing the lac-repressor fused to YFP, this *HO* cut-site is marked by a yellow dot in the nucleus as it is adjacent to an array of 256 copies of *lacO* (Figure 6A). This allows a Rad52 focus formed at the induced DSB to be distinguished from a focus at a spontaneous lesion. With wild-type cells, approximately 60% of the cells contain a Rad52 focus. This number roughly represents the induction efficiency, i.e., the fraction of cells where a DNA DSB was induced, as spontaneous foci are rare in a population of wild-type cells. Of the cells that contained a Rad52 focus after induction, 90% contained a Rad52 focus that co-localized with the induced DNA DSB. This confirms our previous observation that Rad52 is efficiently recruited to this break [39]. With *rad52-C180A-CFP* strains, Rad52-C180A-CFP foci co-localized with the labeled DNA DSB in 55% of the cases, showing that Rad52-C180A is also recruited to a defined DNA DSB. At first glance, it may seem that the recruitment efficiency of Rad52-C180A-CFP is somewhat reduced compared to wild-type Rad52-CFP. However, it is important to note that, unlike in wild-type, many *rad52-C180A-CFP* cells already contain a spontaneous focus before induction. If the DSB induction efficiency in the mutant is similar to that in wild-type (60%), a significant number of *rad52-C180A-CFP* cells containing a spontaneous focus may in fact not have received a DSB during induction. We therefore believe that 55% is an underestimate of the recruitment efficiency as these cells contribute to increase the total number of cells containing a focus after induction. As previously shown for wild-type strains, fortuitous co-localization of Rad52-C180A-CFP and the HO-induced break is less than 5% in control cells that do not express the HO-endonuclease and in cells where the HO-inducible DSB and the *lacO* array are placed on different chromosomes (Figure 6B and 6C).

**Figure 7:**
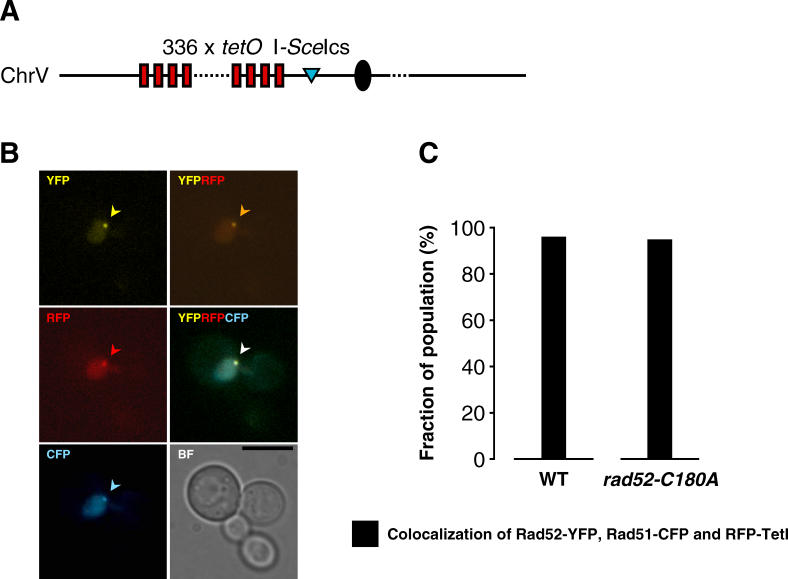
Rad51-CFP and Rad52-C180A-YFP Are Recruited to a Specific DNA DSB (A) Assay in Chromosome V for visualizing an I-*Sce*I-endonuclease inducible DSB. Red boxes: *tetO* sites. Cyan triangle: I-*Sce*I cut-site (I-*Sce*Ics). Solid circle: centromere. (B) Localization of Rad51-CFP and Rad52-C180A-YFP foci to an I-*Sce*I-endonuclease-induced DSB. The panels show YFP, CFP, RFP, RFP/YFP-merged, and CFP/RFP/YFP-merged, as well as a bright field image of representative cells containing Rad51-CFP and Rad52-C180A-YFP in a strain with a *tetO* tandem array next to an I-*Sce*Ics on Chromosome V. The *tetO* tandem array is visualized by TetI-RFP as a red focus. The Rad51-CFP, Rad52-YFP, and TetI-RFP foci are marked by arrowheads. Scale bar, 3 μm. (C) Quantitative analysis of co-localization between Rad52-C180A-YFP/RFP–TetI foci and Rad51-CFP foci. The wild-type dataset is shown for comparison.

Next, we investigated whether Rad51, which is required in the subsequent steps of the repair pathway, is also recruited to a specific DNA DSB in *rad52-C180A* strains. Specifically, co-localization of Rad52-C180A-YFP and Rad51-CFP was determined at a specific DNA DSB induced by the I-*Sce*I endonuclease. In a strain expressing the tetI-repressor fused to RFP (red fluorescent protein), this I-*Sce*I cut-site is marked by a red dot in the nucleus as it is adjacent to an array of 336 copies of *tetO* (Figure 7). Similar to above, approximately 55% of the wild-type cells contained a Rad52-YFP focus after induction. Of these cells, 95% contain a Rad52 focus that co-localized with the marked I-*Sce*I cut-site. Previously, Rad51 and Rad52 have been shown to co-localize at an HO-induced DSB at the *MAT* locus [42]. In agreement with this, we observed that the Rad52 foci that co-localized with an I-*Sce*I cut-site also co-localized with a Rad51-CFP focus in 95% of the cases (Figure 7), demonstrating the presence of both the repair proteins at the I-*Sce*I-induced DSB. Of the 56% *rad52-C180A-YFP* cells that contained a Rad52-C180A-YFP focus, 46% of these foci co-localized with the I-*Sce*I cut-site. Similar to wild-type cells, 94% of the observed Rad52-C180A-YFP foci that co-localized with an I-*Sce*I cut-site also co-localized with a Rad51-CFP focus. These results show that Rad51 is efficiently recruited to a repair focus at a defined DNA DSB in a *rad52-C180A* strain.

### Homologous Recombination Is Efficiently Induced by UV-Irradiation in *rad52* Class C Mutant Strains

The fact that *rad52* class C mutant strains fail to repair DSBs, yet efficiently produce recombinants by HR, prompted us to look for another type of lesion that could induce HR in these mutants. It is well known that HR is stimulated by UV-irradiation [11], which produces DNA lesions that mostly consist of pyrimidine dimers and pyrimidine adducts [12]. To investigate whether UV-rays can induce HR in *rad52* class C mutant strains, the mutants were irradiated by a dose that resulted in 91% viability for wild-type and 15% for *rad52*Δ strains (Table 4). Although rare, DSBs may form as the result of bi-stranded and clustered UV-ray-induced damage [43] and the higher lethality observed for *rad52*Δ strains compared to wild-type strains may be due to the failure of *rad52*Δ strains to repair these DSBs. In agreement with this view, the UV sensitivities measured for *rad52* class C mutant strains, which also fail to repair DSBs, are similar to that obtained for *rad52*Δ cells. At this UV dose, the frequency of heteroallelic interchromosomal HR is increased 410-fold in wild-type strains compared to the spontaneous HR rate (Table 4). Similar stimulations of HR, 230-fold *(rad52-R70A)* to 800-fold *(rad52-T163A),* relative to the spontaneous rates of HR, were observed for all *rad52* class C mutant strains. Importantly, the absolute HR frequencies obtained for most of the *rad52* class C mutant strains after UV irradiation are higher (3.4-fold in the case of *rad52-Y66A*) than the HR frequency obtained with wild-type strains (Table 4). We note that prototroph formation is also strongly stimulated in *rad52*Δ strains. Such prototrophs, which are formed independently of Rad52, do not contribute significantly to the number of prototrophs obtained in wild-type and in class C mutant strains, as they occur at a frequency that is more than 100-fold lower than in these strains (Table 4). Rad52 independent prototroph formation has previously been observed in *rad52*Δ strains designed to detect heteroallelic HR [44]. However, in that study they were accounted for as being generated by UV-induced mutation rather than by HR. Since *rad52* is a known mutator [45], we investigated the ability of diploid wild-type, *rad52-C180A,* and *rad52Δ* strains, which are homozygous for either *leu2-ΔBstE*II or *leu2-ΔEco*RI, for their ability to revert and form prototrophs after UV-irradiation. In all cases, no prototrophs were obtained when similar numbers of cells were plated. Hence, in the case of wild-type and *rad52-C180A,* reversion rates are at least three orders of magnitude lower than the rates of prototroph formation found for heteroalleles. For *rad52Δ* strains it is at least 6-fold reduced. Hence, prototrophs obtained from heterozygous *leu2-ΔBstE*II/*leu2-ΔEco*RI strains after UV irradiation are likely true recombinants. Together, these results show that some UV-induced DNA lesions are substrates for Rad52 class C mutant species in a process that yields viable recombinants at wild-type levels.

**Table 4:**
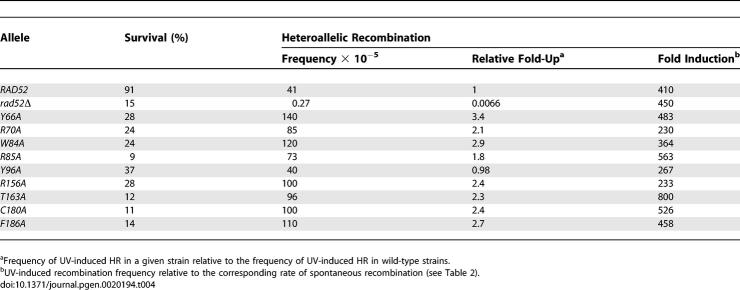
UV-Ray Survival and Induction of Mitotic Heteroallelic Inter-Chromosomal Recombination in *rad52* Mutant Strains

## Discussion

In this study we have thoroughly characterized *rad52* class C mutants, which were originally identified in a plasmid-based screen as being γ-ray sensitive but proficient for HR [17]. First, we confirmed this separation-of-function phenotype in strains where the mutations were integrated into the *RAD52* locus. Next, we examined the separation-of-function phenotype in more detail. With respect to HR, we showed that the mitotic HR observed in *rad52* class C mutants is not due to a compensatory effect of Rad59. With respect to the inability of *rad52* class C mutants to repair DNA DSBs, we analyzed them in three different types of DSB repair assays, one measuring repair by SSA (direct-repeat recombination assay) and two measuring repair by gene conversion (mating-type switching and plasmid gap-repair assays) and firmly established that these mutants are defective in DSB repair by HR. Indeed, the repair efficiencies of the three different types of DSBs in the *rad52* class C mutants resemble that obtained in the absence of Rad52. Moreover, the results obtained with the plasmid gap-repair assay, where the individual contributions of HR and NHEJ to DSB repair can be evaluated, show that a gapped plasmid is repaired preferentially by NHEJ in *rad52* class C mutant strains rather than by HR as in wild-type strains. Thus, we conclude that *rad52* class C mutants fail to repair endonuclease-induced DSBs via mechanisms that require strand invasion of an intact homologous sequence as well as via a more simple mechanism where the ends can be joined by annealing.

Several of the Rad52 functions in DNA DSB repair could potentially be impaired by the *rad52* class C mutations as Rad52-mediated DNA DSB repair is a multi-step reaction that involves many activities, including binding to Rad51, Rad59, RP-A, and DNA [46–50]. All nine *rad52* class C mutations are situated in the evolutionarily conserved N-terminus of Rad52, which contains a DNA-binding domain, domains responsible for Rad52 self-association, and Rad59 binding [50–53]. However, an inspection of the three-dimensional crystal structure of an N-terminal fragment of human Rad52 [52,54] showed that eight of the corresponding amino acid residues in the human Rad52 structure are located in, or close to, the putative DNA-binding groove (see Figure S2). The remaining amino acid residue, HsRad52-Y81 (ScRad52-Y96A) is buried beneath this groove. Moreover, four of the corresponding human Rad52 mutant species: HsRad52-Y51A (ScY66A), HsRad52-R55A (ScR70A), HsRad52-R70A (ScR85A), and HsRad52-Y81A (ScY96A) have been purified, and the three latter species show decreased affinity for single-stranded DNA [52,55]. Hence, the failure of Rad52 class C mutants to perform efficient DSB repair may likely be a consequence of impaired DNA-binding activity.

Defective DNA-binding of Rad52 may affect several stages of the DNA repair process, e.g., resection, homology search, strand invasion, and second-strand capture. The genomic blot analyses indicate that the nucleolytic processing of DSB ends is intact in *rad52* class C mutant strains. This is not surprising, as most models for DSB repair predict that the single-stranded DNA tails at the break are covered by RP-A before Rad52 is recruited [56–59]. In agreement with this view, we have previously shown that RP-A is recruited to a DSB in the absence of Rad52 [60]. In fact, if Rad52 class C mutants are defective in DNA binding, the inability to repair DNA DSBs could simply be explained by the failure of the mutant proteins to recognize and bind to these lesions. In contradiction to this view, we observed in two independent experiments that Rad52-C180A was recruited to defined DNA DSBs, one induced by the HO-endonuclease on Chromosome III and one induced by I-*Sce*I on Chromosome V. These results indicate that the DNA-binding domain in the Rad52 N-terminus is not required for Rad52 recruitment to the DNA lesion. This recruitment is then most easily explained by a scenario where Rad52 is attracted to the DNA lesion via its ability to interact with RP-A when the latter has formed a complex with single-stranded DNA at the DSB. This view is supported by the observation that Rad52-C180A, like wild-type Rad52, physically interacts with Rfa1 in a two-hybrid assay (unpublished data) and that no wild-type Rad52 focus is formed at a lesion in the absence of RP-A [60]. Accordingly, *rad52* class C mutations affect a function in Rad52 that is downstream of damage recognition. In fact, we have shown that the reaction is blocked after Rad51 has been recruited to the DNA DSB. However, our experiments do not show whether Rad51 or Rad52 are bound to the damaged DNA in the repair center. The possibility exists that they are just attracted to the lesion via protein-protein interactions. In this case, the failure of Rad52 class C mutants to efficiently bind DNA could result in its inability to mediate the replacement of RP-A by Rad51, thus impairing repair. If, on the other hand, a Rad51 filament is formed at the lesion in *rad52* class C mutant cells, we speculate that the DSB remains unrepaired either because the defective Rad52 DNA-binding activity impairs a Rad52-catalyzed homology search important for strand invasion or an annealing step important for second-strand capture. The latter view may explain the observation that the BIR efficiency is reduced only 3-fold in *rad52-C180A* strains as second-strand capture is not required in BIR.

It is generally believed that DSBs are the lesions that initiate spontaneous HR. However, here we show that nine *rad52* class C mutants are proficient for spontaneous inter- and intrachromosomal heteroallelic HR, but fail to repair different types of DSBs. Since Rad52 forms spontaneous repair foci during S-, but not during the G1-phase of the cell cycle [38], an alternative source of spontaneous HR could be recombinogenic DNA ends generated when a migrating replication fork converts a nick into a DSB. Such ends may be easier to repair than those induced by γ-irradiation and endonucleases as they require only a one-ended invasion of the intact strand to restore the replication fork. However, *rad52* class C mutant strains are sensitive to camptothecin and Top1-T722A expression. If the replication-induced ends in these experiments are equivalent to the DNA end rescued in the BIR experiment, these results may appear surprising as BIR is reduced only 3-fold in *rad52*-C180A compared to wild-type. However, unlike in the BIR experiment, multiple lesions are likely produced when Top1-T722A is expressed and in the presence of camptothecin, and strains with a weakened, but not abolished, ability to repair such lesions may die. The possibility therefore still remains that one-ended DNA breaks contribute to spontaneous HR. However, if this contribution was large, one would expect that even a small, but significant, reduction of the efficiency of one-ended DNA break repair should be reflected as reduced HR levels. Since this is not the case in the *rad52* class C mutants, replication-induced breaks are likely to initiate only a minor fraction of the spontaneous recombination events.

Based on the above, we find it unlikely that DSBs contribute substantially to the spontaneous HR observed for *rad52* class C mutant strains, since mutant cells only rarely survive even a single DSB. This view is supported by work from Fabre and colleagues based on their studies of *srs2* and *sgs1* strains [61]. They argued that *srs2 sgs1* synthetic lethality is due to a toxic recombination intermediate. If this intermediate were initiated by spontaneous DSBs, they must occur at a sufficiently high frequency to prevent propagation of *srs2 sgs1* strains. However, since *rad52 null lig4* as well as *rad52 null lig4 srs2 sgs1* strains, which cannot repair DSBs (i.e., no HR and no NHEJ), are viable, they conclude that DSBs cannot be initiating the frequent recombination intermediates that kill *srs2 sgs1* strains. In this context, it is important to note that around 75% of *rad52-C180A* cells spontaneously develop a Rad52-C180A focus during the cell cycle. If these foci solely represent attempts to repair DSBs in the genome, then *rad52-C180A* strains should be inviable due to their inability to repair DSBs. Moreover, we observe that Rad52-C180A foci are turned over before cell division, albeit at a slow rate, suggesting that repair of the spontaneous lesions is in fact completed.

We also note that some of the *rad52* class C mutants are hyperrecombinogenic. This behavior is similar to mutations in proteins of the Mre11-Rad50-Xrs2 complex (MRX), which act at an early stage of both HR and NHEJ [3], and also produce a hyper-recombination phenotype [62]. The high HR rate in these MRX mutants is thought to be due to a shift in the preferred repair template from the sister chromatid to the homologous chromosome, hence increasing the number of scorable recombinants [63]. We find that the median lifetime of a Rad52 class C mutant repair focus is seven times longer than a wild-type Rad52 focus. This longer time frame of repair may increase the frequency of genetic exchange with the homolog. Alternatively, more recombinational lesions may be formed in *rad52* class C mutant strains. In fact, we observe that a larger number of cells spontaneously form Rad52 repair foci during the cell cycle in *rad52* class C mutant cells than in wild-type cells.

If only a minor fraction of spontaneous HR is initiated by DSBs, alternative lesions need to be considered as triggers for HR. In many of the original models for HR, DNA nicks and single-stranded gaps were proposed to initiate HR [64–66]. In the present study, we find that UV-irradiation leads to a dramatic increase in interchromosomal heteroallelic HR in *rad52* class C mutant strains. This is similar to what has been observed for wild-type strains. UV-rays mostly produce pyrimidine dimmers, and in the dark, the majority of these lesions are repaired by the nucleotide excision and base excision repair pathways. This type of repair produces nicks and single-stranded DNA gaps that could be recombinogenic. Moreover un-repaired pyrimidine dimers may lead to stalled replication forks and expose regions of single-stranded DNA. Single-stranded gaps are potent substrates for recombination in E. coli via the RecFOR pathway [13–16], and it is interesting to note that both the RecFOR complex and Rad52 mediate replacement of a single-strand binding protein, SSB and RP-A, respectively, to allow access of a protein with a strand invasion activity, RecA and Rad51, respectively, during DNA repair [16,56,57,59,67–69]. In addition, a stalled replication fork may produce a DNA substrate suitable for HR, if the fork is regressed into a “chicken foot” structure and the resulting DNA end processed by nucleases to produce a stretch of single-stranded DNA [30]. Considering that Rad52 repair foci form during DNA replication, such lesions are attractive candidates as substrates that elicit spontaneous HR.

Recently, it was demonstrated that nicked intermediates produced by mutant RAG proteins during V(D)J recombination can be channeled into HR [70]. Furthermore, the spectrum of spontaneous recombinants obtained in an assay that measures direct-repeat gene conversion and unequal sister-chromatid exchange in a mammalian cell line is similar to the spectrum obtained after addition of camptothecin, but different from the spectrum obtained after induction of recombination by the endonuclease I-*Sce*I [71]. Accordingly, lesions other than DSBs may also play a significant role in spontaneous HR in higher eukaryotes.

## Materials and Methods

### Genetic methods, strains, and plasmids.

All media were prepared as described previously [72] with minor modifications as the synthetic medium contains twice the amount of leucine (60 mg/L). Standard genetic techniques were used to manipulate yeast strains [73] and transformations were performed according to [74]. All strains are derivatives of W303 [75] except that they are *RAD5* [76,77] and are listed in Table S1. Plasmids pJH283 [78] and pJH132 contain a GAL10::*HO* fusion in a *CEN4 ARS1*-based vector as well as a *TRP1* and a *URA3* marker for selection, respectively, and were kindly provided by J. Haber. For construction of pRS413-*TRP1,* a replicative *ARSH4/CEN6*-based plasmid, see Protocol S1. The plasmid CFV/D8B-tg was a kind gift from Dr. L. Symington.

### Viability after γ- and UV-irradiation, HO-endonuclease induction, and exposure to camptothecin sensitivity.

HO-endonuclease induction and γ- and UV-irradiation was performed as previously described [27,79], except UV-irradiation was performed by using a Stratalinker 2400 UV Crosslinker from Stratagene (La Jolla, California, United States) and cells were exposed to a dose of 50 J/m^2^ at 254 nm. The dose rate of the γ-irradiator was 2.1 krad/min. The slope (α) of the resulting straight line can be used to calculate an LD37 value -ln(1/0.37)/lnα. The LD37 value represents the dose in krad necessary to induce a mean of one lethal hit per cell and was used to quantitatively compare survival of different strains. For details see Protocol S1. Camptothecin sensitivity was assayed by growing cells overnight to mid-log phase. A 10-fold serial dilution for each culture was made and spotted on two individual YPD plates containing 0 and 0.5 μg camptothecin, respectively. The ability of each strain to form colonies on each plate was evaluated after 3 d incubation at 30 °C.

### Determination of spontaneous and induced mitotic recombination rates.

Spontaneous mitotic HR between *leu2-ΔEco*RI and *leu2-ΔBst*EII heteroalleles was measured in diploid strains (interchromosomal HR) or in haploid strains (intrachromosomal HR) as previously described [27,79]. The intrachromosomal HR assay used contains the *leu2-*heteroalleles in the proximal configuration. HO-endonuclease-induced intrachromosomal direct-repeat HR was performed as previously described [27,79]. UV-induced interchromosomal *leu2-ΔEco*RI/*leu2-ΔBst*EII heteroallelic HR experiments were made in triplicates for each strain analyzed. The HR frequency after UV-irradiation was determined by dividing the total number of recombinants in the culture by the total corresponding number of surviving cells following irradiation.

### Determination of BIR efficiency.

The ability of selected strains to perform BIR was evaluated by a chromosome fragmentation assay [36,37] . Specifically, 1 μg of either intact or *Sna*BI linearized CFV/D8B-tg plasmid was transformed into relevant *ura3–1, ade2–1* strains. The BIR efficiency for each strain was determined as the number of BIR transformants obtained by linearized CFV/D8B-tg divided by the number of transformants obtained by uncut CFV/D8B-tg. BIR transformants were identified as Ura+ transformants with a low rate of red sectoring, in contrast to transformants resulting from plasmids that had simply re-circularized, which were characterized by a very high rate of red sectoring. For further details, see [36].

### Plasmoduction.

Details of the plasmoduction protocol for transferring plasmids into haploid strains by mating will be published elsewhere (RJDR and RR, unpublished data). In brief, the *MAT*α plasmid donor strain J1361 was transformed with pWJ1439 *(TOP1),* pWJ1440 *(top1-T722A),* or pRS415 (vector), and crossed to the *MAT* **a** *rad52* class C mutants using a mating reaction that predominantly produces heterokaryons rather than diploids. Plasmid transfer into the recipient nucleus is selected for, while counter-selection is applied to the donor nucleus using 5-FOA and galactose. The selection plates were photographed after 3 d incubation at 30 °C to measure the growth of the recipient *MAT* **a** strains containing the plasmids.

### Gap-repair assay.

To evaluate the gap-repair frequency, strains were transformed with circular and linear pRS413-*TRP1.* The linear substrate was made by cutting the *TRP1* marker in pRS413-*TRP1* with *Bsg*I and *Mfe*I. The resulting gap spans the region that harbors the *trp1–1* amber stop-codon mutation [80] in the genome. Transformants containing a plasmid sealed by HR or by NHEJ is therefore His+ Trp-. The repair frequency was calculated by dividing the number of His+ Trp- transformants with the number of transformants obtained in a parallel experiment using circular pRS413-*TRP1.* To determine whether the plasmid was sealed by NHEJ or by HR, a PCR assay using plasmid specific primers, T3 and T7 (5′-AATTAACCCTCACTAAAGGG-3′ and 5′-TAATACGACTCACTATAGGG-3′) was employed. Events generated by NHEJ and HR produces PCR fragments of approximately 902 bp and 1,175 bp, respectively. If the 1,175-bp fragment is generated by HR it contains *trp1–1.* This was verified by demonstrating the absence of a *Bsu36I* site in the 1,175-bp fragment.

### Determination of Rad52 concentrations in RAD52 and *rad52* strains.

Western blot analysis and subsequent quantification of band intensities were performed as previously described [50] except for minor modifications (see Protocol S1).

### Yeast live cell imaging and fluorescence microscopy.

Cells from liquid cultures were imaged by fluorescence microscopy as described previously [38,60]. Image acquisition times for Rad52-CFP and Rad52-YFP were 750 ms and 1,000 ms, respectively. Induction of Rad52-CFP and Rad52-YFP foci by γ-irradiation was done after the cells received a dose of 80 krad followed by 30 min of recovery in liquid SC medium at 23 °C. The fluorescently marked (YFP-LacI) chromosomal *HO* cut-site, the fluorescently marked (RFP-TetI) chromosomal I-*Sce*I cut-site, and induction of DSBs at these sequences by the HO- and I-*Sce*I endonucleases, respectively, were described previously [39]. The red fluorophore used in this study is the monomeric version of DsRed (mRFP1; [81]). The yellow- and blue-shifted enhanced variants of the GFP gene and the DNA sequence encoding the monomeric version of DsRed (mRFP1) were generous gifts from R. Tsien (University of California, San Diego, California, United States).

### Statistical methods.

A Student's *t*-test was used to determine the significance of differences among the mutants versus wild-type and *rad52*Δ strains when comparing protein levels and mitotic and direct-repeat HR rates. For replacement events, the test of significance was determined using a *chi*-square analysis.

## Supporting Information

Figure S1 Induced Mating-Type Switching Is Lethal in *rad52* Class C Mutant Strains (392 KB DOC)

Figure S2 *rad52* Class C Mutations Are Located at the DNA-Binding Site of Rad52 (4.2 MB DOC)

Protocol S1 Supporting Protocol (48 KB DOC)

Table S1 Strains Used in This Study (84 KB DOC)

## Abbreviations

BIR: break-induced replication
CFP: cyan fluorescent protein
DSB: double-strand break
HR: homologous recombination
NHEJ: non-homologous end-joining
SSA: single-strand annealing
YFP: yellow fluorescent protein
