# Supplementary material for: The Role of DNA Double-Strand Breaks in Spontaneous Homologous Recombination in S. cerevisiae
Source: PLoS Genet. 2006 Nov 10;2(11):e194. doi: 10.1371/journal.pgen.0020194 (PMC1635536; doi:10.1371/journal.pgen.0020194)
Supplement: Supplementary file 1 [file pgen.0020194.sd001.doc]

**Supporting Protocols**

**Construction of pRS413-*TRP1*.** A PCR fragment containing the entire *TRP1* gene was generated by primers SalI_TRP1_fw (TGGATGGTgtcgacGATTGTACTGAGAGTGCACC) and EcoRI_TRP1_Rv (ACCTTTACgaattcGCATAGGCAAGTGCACAAAC) using pRS414 [1] as substrate. The resulting fragment was trimmed by digestion with *Eco*RI and *Sal*I and inserted into an *Eco*RI-*Sal*I fragment of pRS413 [1], a *CEN4* vector that contains a *HIS3* selectable marker, to produce pRS413-*TRP1*.

**Construction of strains.** All class C mutant alleles were transferred into the *RAD52* locus in W1588-4C from their original pWJ1086 context, see [2] by a PCR based method [3]. The *rad52* sequence in all *rad52* class C mutant strains were extended in frame by the gene encoding CFP or YFP. This was done by transforming the mutant strains by plasmid pWJ1385, which is a pRS414 based plasmid that contains the structural gene of *RAD52* under the control of the *MET25* promoter and terminator. Subsequently, endogenous *rad52* mutant genes were tagged by CFP or YFP sequences according to the method described in [4].

**Viability after γ- and UV-irradiation, *HO*-endonuclease induction and exposure to camptothecin sensitivity.** γ-irradiation was performed as previously described [5] using a Gammacell-220 ^60^Co irradiator (Atomic Energy of Canada). UV irradiation was performed by using a Stratalinker® 2400 UV Crosslinker from Stratagene. Cells were exposed to a dose of 30 J/m^2^ at 254nm. Triplicates were performed for all experiments and all strains were sonicated prior to plating. For each strain an appropriate number of cells were plated on YPD plates (γ-ray experiments) or SC plates (UV-ray experiments) before and after irradiation. Viability was determined after three days of incubation in the dark at 30°C by dividing the number of colony forming units in a culture after irradiation with the corresponding number obtained before irradiation. To evaluate the ability of *rad52* mutant strains to survive sustained expression of the *HO*-endonuclease in strains containing the native *MAT* locus, selected strains were transformed by the *HO-*endonuclease encoding plasmid pJH283. For each strain, at least two of the resulting transformants were grown overnight in SC-Ura to midlog phase and each culture spotted in ten-fold serial dilutions on two plates of solid SC-Ura medium containing either glucose or galactose as carbon source, respectively. The ability of *rad52* strains, which contain a single *HO*cs either at the *MAT* locus or in the context of a direct repeat, *leu2-∆Eco*RI*::URA3-HOcs::leu2-∆Bst*EII, to survive a brief expression of the *HO*-endonuclease was determined by transforming strains by pJH283 or pJH132, respectively. Strains were pre-grown in either SC-Ura 2% raffinose or SC-Trp 2% raffinose, transferred to liquid SC-Ura, 2% galactose or SC-Trp, 2% galactose and incubated for 1 or 1.5 hours. The strains were appropriately diluted and plated on solid YPD medium before and after induction and the plates were incubated for 2 days at 30° before the colonies were counted. In the case of surviving a cut at the *MAT* locus, viability was determined by dividing the number of Ura+ colonies with the number of Ura+ colonies before induction. In the case of surviving a cut between directly repeated *leu2* heteroalleles, viability was determined by dividing the number of Trp+ colonies with the number of Trp+ colonies before induction. The scheme to monitor intermediates in DSB repair of a *HO*-endonuclease induced break situated between *leu2* heteroalleles by Southern blot analysis was described previously [6]. DNA was visualized using phosphorImager 445 SI and quantified by ImageQuant software (molecular Dynamics). A probe detecting the *ADE2* locus was added as a loading control. This probe was generated by the primers ADE-F: 5’-CTCACTGGCTTGTTCCACAGG and ADE-B: 5’-ATGGCTCCTTTTCCAATCCTC.

***HO*-endonuclease and UV induced recombination.** To evaluate the ability of *rad52* mutant strains to perform *HO*-endonuclease induced mating-type switching, the colonies obtained on YPD plates obtained from cultures that were briefly exposed to inductive medium, see above, were replicated to SC-Ura plates and to plates containing lawns of *MAT***a** (D297-4B or R113) and *MAT*α tester strains (B3847 or R194). The mating-type switching efficiency was determined by dividing the number of Ura+ *MAT*α colonies with the total number of Ura+ colonies. The numbers are means of five to seven individual trials for each *rad52* mutant strain. To evaluate *HO*-endonuclease induced direct-repeat HR, colonies on YPD plates obtained from cultures that were briefly exposed to inductive medium were replica plated to SC-Leu, SC-Ura and SC-Trp plates to detect HR events (Leu+ cells) and deletion events (Ura- cells) and to determine survival rates (Trp+ cells) see above. The numbers are means of five independent trials.

In UV-induced interchromosomal *leu2-∆Eco*RI/*leu2-∆Bst*EII heteroallelic HR experiments, triplicates were made for each strain analyzed. An appropriate number of cells were plated onto SC and SC-Leu plates and irradiated as described above. The HR frequency after UV irradiation was determined by dividing the total number of recombinants (Leu+ cells) in the culture by the corresponding total number of surviving cells following irradiation.

**Determination of Rad52 concentration in *RAD52* and *rad52* strains.** Three different 10 ml cultures of each strain were grown to mid log phase. The total protein extraction was done by glass bead disruption as described in [7]. The protein was separated on sodium dodecyl sulfate (SDS)-10% polyacrylamide gel and electrically transferred to Immobilon-P membrane (Millipore). The membrane was blocked by 45 ml nonfat dry milk and probed with Rad52 antibody [8] that has been affinity purified using a Rad52-Sepharose column [8]. The resulting immunocomplexes was visualized using an enhanced chemiluminescence kit (Amersham) and analyzed by ImageQuant (Molecular Dynamics). The relative protein levels (three trials for each allele) are given as percentages of the Rad52 level in wild-type strains.

References

1.	Sikorski RS, Hieter P (1989) A system of shuttle vectors and yeast host strains designed for efficient manipulation of DNA in *Saccharomyces cerevisiae*. Genetics 122: 19-27.

2.	Mortensen UH, Erdeniz N, Feng Q, Rothstein R (2002) A molecular genetic dissection of the evolutionarily conserved N terminus of yeast Rad52. Genetics 161: 549-62.

3.	Erdeniz N, Mortensen UH, Rothstein R (1997) Cloning-free PCR-based allele replacement methods. Genome Res 7: 1174-83.

4.	Lisby M, Rothstein R, Mortensen UH (2001) Rad52 forms DNA repair and recombination centers during S phase. Proc Natl Acad Sci U S A 98: 8276-82.

5.	Smith J, Rothstein R (1995) A mutation in the gene encoding the *Saccharomyces cerevisiae* single-stranded DNA-binding protein Rfa1 stimulates a *RAD52*-independent pathway for direct-repeat recombination. Molecular & Cellular Biology 15: 1632-41.

6.	Bai Y, Davis AP, Symington LS (1999) A novel allele of *RAD52* that causes severe DNA repair and recombination deficiencies only in the absence of *RAD51* or *RAD59*. Genetics 153: 1117-30.

7.	Harlow E, Lane D (1988) Antibodies: A Laboratory Manual. Cold Spring Harbor: Cold Spring Harbor Laboratory.

8.	Mortensen UH, Bendixen C, Sunjevaric I, Rothstein R (1996) DNA strand annealing is promoted by the yeast Rad52 protein. Proceedings of the National Academy of Sciences of the United States of America 93: 10729-34.
