# Supplementary material for: The Role of DNA Double-Strand Breaks in Spontaneous Homologous Recombination in S. cerevisiae
Source: PLoS Genet. 2006 Nov 10;2(11):e194. doi: 10.1371/journal.pgen.0020194 (PMC1635536; doi:10.1371/journal.pgen.0020194)
Supplement: Supplementary file 2 [file pgen.0020194.sg001.doc]

**Figure S1.** Induced mating-type switching is lethal in *rad52* class C mutant strains

Strains were transformed with a plasmid that allowed galactose induced expression of the *HO*-endonuclease. A spot assay was performed to evaluate the sensitivity of *rad52* strains to expression of the *HO*-endonuclease. Serial 10-fold dilutions of wild-type, *rad52-C180A* and *rad52*Δ strains were spotted on solid repressive medium, SC-Ura + 2% glucose and solid inductive medium, SC-Ura + 2% galactose as indicated.
