# Supplementary material for: The Role of DNA Double-Strand Breaks in Spontaneous Homologous Recombination in S. cerevisiae
Source: PLoS Genet. 2006 Nov 10;2(11):e194. doi: 10.1371/journal.pgen.0020194 (PMC1635536; doi:10.1371/journal.pgen.0020194)
Supplement: Supplementary file 3 [file pgen.0020194.sg002.doc]

**Figure S2.** *rad52* class C mutations are located at the DNA binding site of Rad52

A single subunit of the human Rad52 N-terminal fragment (amino acid residues 1-212) is shown in upper panels (A and B). The positions of amino acid residues corresponding to mutated residues in *S. cerevisiae* class C *rad52* mutations are presented in yellow and indicated with arrows. Representations of Rad52 subunits (N-terminal fragments) in an undecameric ring are given in lower panels (C and D). Rad52 coordinates are from Kagawa et al. [1] and from Singleton et al. [2].

References

1.	Kagawa W, Kurumizaka H, Ishitani R, Fukai S, Nureki O, Shibata T, Yokoyama S (2002) Crystal structure of the homologous-pairing domain from the human Rad52 recombinase in the undecameric form. Mol Cell 10: 359-71.

2.	Singleton MR, Wentzell LM, Liu Y, West SC, Wigley DB, Kagawa W, Kurumizaka H, Ishitani R, Fukai S, Nureki O, Shibata T, Yokoyama S (2002) Structure of the single-strand annealing domain of human RAD52 protein. Proc Natl Acad Sci U S A 99: 13492-7.
