# Supplementary material for: The Role of DNA Double-Strand Breaks in Spontaneous Homologous Recombination in S. cerevisiae
Source: PLoS Genet. 2006 Nov 10;2(11):e194. doi: 10.1371/journal.pgen.0020194 (PMC1635536; doi:10.1371/journal.pgen.0020194)
Supplement: Supplementary file 4 [file pgen.0020194.st001.doc]

| **Table S1.** Strains used in this study. All strains are made in this study with the exception of the strains marked by footnotes | | |
| --- | --- | --- |
| Strain^a^ | | Genotype |
| W1588-4C^b^ | *MAT***a** *ade2-1 can1-100 his3-11,15 leu2-3,112 trp1-1 ura3-1* | |
|  |  | |
| J787 | *MAT***a** *rad52-Y66A ade2-1 can1-100 his3-11,15 leu2-3,112 trp1-1 ura3-1* | |
| J788 | *MAT***a** *rad52-R70A ade2-1 can1-100 his3-11,15 leu2-3,112 trp1-1 ura3-1* | |
| J774 | *MAT***a** *rad52-W84A ade2-1 can1-100 his3-11,15 leu2-3,112 trp1-1 ura3-1* | |
| J738 | *MAT***a** *rad52-R85A ade2-1 can1-100 his3-11,15 leu2-3,112 trp1-1 ura3-1* | |
| J765-1 | *MAT***a** *rad52-Y96A ade2-1 can1-100 his3-11,15 leu2-3,112 trp1-1 ura3-1* | |
| J771 | *MAT***a** *rad52-R156A ade2-1 can1-100 his3-11,15 leu2-3,112 trp1-1 ura3-1* | |
| J783 | *MAT***a** *rad52-T163A ade2-1 can1-100 his3-11,15 leu2-3,112 trp1-1 ura3-1* | |
| J786 | *MAT***a** *rad52-C180A ade2-1 can1-100 his3-11,15 leu2-3,112 trp1-1 ura3-1* | |
| J777 | *MAT***a** *rad52-F186A ade2-1 can1-100 his3-11,15 leu2-3,112 trp1-1 ura3-1* | |
|  |  | |
| W3749-14C^c^ | *MAT***a** *RAD52-YFP ADE2 bar1::LEU2 can1-100 his3-11,15 leu2-3,112 trp1-1 ura3-1* | |
| J1207 | *MAT***a** *rad52-Y66A-YFP ADE2 bar1::LEU2 can1-100 his3-11,15 leu2-3,112 trp1-1 ura3-1* | |
| W3840-15D | *MAT***a** *rad52-R70A-YFP ADE2 bar1::LEU2 can1-100 his3-11,15 leu2-3,112 trp1-1 ura3-1* | |
| UM190-6B | *MAT***a** *rad52-W84A-YFP ADE2 bar1::LEU2 can1-100 his3-11,15 leu2-3,112 trp1-1 ura3-1* | |
| W3734-8B | *MAT***a** *rad52-R85A-YFP ADE2 bar1::LEU2 can1-100 his3-11,15 leu2-3,112 trp1-1 ura3-1* | |
| UM140-2A | *MAT***a** *rad52-Y96A-YFP ADE2 bar1::LEU2 can1-100 his3-11,15 leu2-3,112 trp1-1 ura3-1* | |
| W3167-5A | *MAT***a** *rad52-R156A-YFP ADE2 bar1::LEU2 can1-100 his3-11,15 leu2-3,112 trp1-1 ura3-1* | |
| W4024-1B | *MAT***a** *rad52-T163A-YFP ADE2 bar1::LEU2 can1-100 his3-11,15 leu2-3,112 trp1-1 ura3-1* | |
| W2594-4A | *MAT***a** *rad52-C180A-8ALA-YFP ADE2 bar1::LEU2 can1-100 his3-11,15 leu2-3,112 trp1-1 ura3-1* | |
| UM31-1D | *MAT***a** *rad52-F186A-YFP ADE2 bar1::LEU2 can1-100 his3-11,15 leu2-3,112 trp1-1 ura3-1* | |
|  |  | |
| W2282-2C | *Mat::HIS3 rad52::HIS5 leu2-*Δ*EcoRI::URA3-HO::leu2-*Δ*BstEII trp1-1* | |
| W2158-1C | *Mat::HIS3 Rad52 leu2-*Δ*EcoRI::URA3-HO::leu2-*Δ*BstEII trp1-1* | |
| J822 | *Mat::HIS3 rad52-Y66A leu2-*Δ*EcoRI::URA3-HO::leu2-*Δ*BstEII trp1-1* | |
| W4158-3D | *Mat::HIS3 rad52-R70A leu2-*Δ*EcoRI::URA3-HO::leu2-*Δ*BstEII trp1-1* | |
| W4159-6C | *Mat::HIS3 rad52-W84A leu2-*Δ*EcoRI::URA3-HO::leu2-*Δ*BstEII trp1-1* | |
| W2158-16B | | *Mat::HIS3 rad52-R85A leu2-*Δ*EcoRI::URA3-HO::leu2-*Δ*BstEII trp1-1* |
| W2177-15D | | *Mat::HIS3 rad52-Y96A leu2-*Δ*EcoRI::URA3-HO::leu2-*Δ*BstEII trp1-1* |
| W4157-5A | | *Mat::HIS3 rad52-R156A leu2-*Δ*EcoRI::URA3-HO::leu2-*Δ*BstEII trp1-1* |
| W2180-3A | | *Mat::HIS3 rad52-T163A leu2-*Δ*EcoRI::URA3-HO::leu2-*Δ*BstEII trp1-1* |
| W2181-8A | | *Mat::HIS3 rad52-C180A leu2-*Δ*EcoRI::URA3-HO::leu2-*Δ*BstEII trp1-1* |
| W2179-16A | | *Mat::HIS3 rad52-F186A leu2-*Δ*EcoRI::URA3-HO::leu2-*Δ*BstEII trp1-1* |
|  | |  |
| J1361 | | *Mat***a** *16X Gal-CEN - K.lactis URA3 ADE2+ kar1*Δ*15 rho+* |
|  | |  |
| W4021-20A | | *Mat::HIS3 ADE2 bar1::LEU2 trp1-1 LYS2 RAD52-C180A-CFP HO-iYCL018W his3-11,15::YFP-LacI::his3-x leu2-3,112::LacO::LEU2* |
| W4341-6D | | *Mat::HIS3 ADE2 bar1::LEU2 trp1-1 LYS2 RAD52-C180A-CFP his3-11,15::YFP-LacI::his3-x leu2-3,112::LacO::LEU2* |
| W4341-16A | | *Mat::HIS3 ADE2 bar1::LEU2 trp1-1 LYS2 RAD52-C180A-CFP HO-iYDR010C his3-11,15::YFP-LacI::his3-x leu2-3,112::LacO::LEU2* |
| UM214-60B  UM215-22B | | *Mat***a** *ade2-1 bar1::LEU2 trp1-1 LYS2 RAD52-YFP RAD51-CFP ura::3xURA3-tetOx112 I-SceI-iYEL023C TetI-mRFP1*  *Mat***a** *ade2-1 bar1::LEU2 trp1-1 LYS2 RAD52-C180A-YFP RAD51-CFP ura::3xURA3-tetOx112 I-SceI-iYEL023C TetI-mRFP1* |
|  | |  |
|  | |  |
|  | |  |
|  | |  |
| UM157 | | *MAT***a** *ade2-1 can1-100 his3-11,15 leu2*∆*BstEII lys2 TRP1 ura3-1*  *MAT*α *ade2-1 can1-100 his3-11,15 leu2*∆*EcoRI LYS2 trp1-1 ura3-1* |
| UM158 | | *MAT***a** *rad59Δ ade2-1 can1-100 his3-11,15 leu2*Δ*BstEII lys2 TRP1 ura3-1*  *MAT*α *rad59Δ ade2-1 can1-100 his3-11,15 leu2*Δ*EcoRI LYS2 trp1-1 ura3-1* |
| UM159 | | *MAT***a** *rad52::HIS5 rad59Δ ade2-1 can1-100 his3-11,15 leu2*Δ*BstEII lys2 TRP1 ura3-1*  *MAT*α *rad52::HIS5 rad59Δ ade2-1 can1-100 his3-11,15 leu2*Δ*EcoRI LYS2 trp1-1 ura3-1* |
| UM160 | | *MAT***a** *rad52-R70A ade2-1 can1-100 his3-11,15 leu2*Δ*BstEII lys2 TRP1 ura3-1*  *MAT*α *rad52-R70A ade2-1 can1-100 his3-11,15 leu2*Δ*EcoRI LYS2 trp1-1 ura3-1* |
| UM161 | | *MAT***a** *rad52-W84A ade2-1 can1-100 his3-11,15 leu2*Δ*BstEII lys2 TRP1 ura3-1*  *MAT*α *rad52-W84A ade2-1 can1-100 his3-11,15 leu2*Δ*EcoRI LYS2 trp1-1 ura3-1* |
| UM162 | | *MAT***a** *rad52-R85A ade2-1 can1-100 his3-11,15 leu2*Δ*BstEII lys2 TRP1 ura3-1*  *MAT*α *rad52-R85A ade2-1 can1-100 his3-11,15 leu2*Δ*EcoRI LYS2 trp1-1 ura3-1* |
| UM163 | | *MAT***a** *rad52-Y96A ade2-1 can1-100 his3-11,15 leu2*Δ*BstEII lys2 TRP1 ura3-1*  *MAT*α *rad52-Y96A ade2-1 can1-100 his3-11,15 leu2*Δ*EcoRI LYS2 trp1-1 ura3-1* |
| UM164 | | *MAT***a** *rad52- R156A ade2-1 can1-100 his3-11,15 leu2*Δ*BstEII lys2 TRP1 ura3-1*  *MAT*α *rad52-R156A ade2-1 can1-100 his3-11,15 leu2*Δ*EcoRI LYS2 trp1-1 ura3-1* |
| UM165 | | *MAT***a** *rad52-T163A ade2-1 can1-100 his3-11,15 leu2*Δ*BstEII lys2 TRP1 ura3-1*  *MAT*α *rad52-T163A ade2-1 can1-100 his3-11,15 leu2*Δ*EcoRI LYS2 trp1-1 ura3-1* |
| UM166 | | *MAT***a** *rad52-C180A ade2-1 can1-100 his3-11,15 leu2*Δ*BstEII lys2 TRP1 ura3-1*  *MAT*α *rad52-C180A ade2-1 can1-100 his3-11,15 leu2*Δ*EcoRI LYS2 trp1-1 ura3-1* |
| UM167 | | *MAT***a** *rad52-F186A ade2-1 can1-100 his3-11,15 leu2*Δ*BstEII lys2 TRP1 ura3-1*  *MAT*α *rad52-F186A ade2-1 can1-100 his3-11,15 leu2*Δ*EcoRI LYS2 trp1-1 ura3-1* |
| UM168 | | *MAT***a** *rad52-Y66A rad59Δ ade2-1 can1-100 his3-11,15 leu2*Δ*BstEII lys2 TRP1 ura3-1*  *MAT*α *rad52-Y66A rad59Δ ade2-1 can1-100 his3-11,15 leu2*Δ*EcoRI LYS2 trp1-1 ura3-1* |
| UM169 | | *MAT***a** *rad52-R70A rad59Δ ade2-1 can1-100 his3-11,15 leu2*Δ*BstEII lys2 TRP1 ura3-1*  *MAT*α *rad52-R70A rad59Δ ade2-1 can1-100 his3-11,15 leu2*Δ*EcoRI LYS2 trp1-1 ura3-1* |
| UM170 | | *MAT***a** *rad52-W84A rad59Δ ade2-1 can1-100 his3-11,15 leu2*Δ*BstEII lys2 TRP1 ura3-1*  *MAT*α *rad52-W84A rad59Δ ade2-1 can1-100 his3-11,15 leu2*Δ*EcoRI LYS2 trp1-1 ura3-1* |
| UM171 | | *MAT***a** *rad52-R85A rad59Δ ade2-1 can1-100 his3-11,15 leu2*Δ*BstEII lys2 TRP1 ura3-1*  *MAT*α *rad52-R85A rad59Δ ade2-1 can1-100 his3-11,15 leu2*Δ*EcoRI LYS2 trp1-1 ura3-1* |
| UM172 | | *MAT***a** *rad52-Y96A rad59Δ ade2-1 can1-100 his3-11,15 leu2*Δ*BstEII lys2 TRP1 ura3-1*  *MAT*α *rad52-Y96A rad59Δ ade2-1 can1-100 his3-11,15 leu2*Δ*EcoRI LYS2 trp1-1 ura3-1* |
| UM173 | | *MAT***a** *rad52- R156A rad59Δ ade2-1 can1-100 his3-11,15 leu2*Δ*BstEII lys2 TRP1 ura3-1*  *MAT*α *rad52-R156A rad59Δ ade2-1 can1-100 his3-11,15 leu2*Δ*EcoRI LYS2 trp1-1 ura3-1* |
| UM174 | | *MAT***a** *rad52-T163A rad59Δ ade2-1 can1-100 his3-11,15 leu2*Δ*BstEII lys2 TRP1 ura3-1*  *MAT*α *rad52-T163A rad59Δ ade2-1 can1-100 his3-11,15 leu2*Δ*EcoRI LYS2 trp1-1 ura3-1* |
| UM175 | | *MAT***a** *rad52-C180A rad59Δ ade2-1 can1-100 his3-11,15 leu2*Δ*BstEII lys2 TRP1 ura3-1*  *MAT*α *rad52-C180A rad59Δ ade2-1 can1-100 his3-11,15 leu2*Δ*EcoRI LYS2 trp1-1 ura3-1* |
| UM176 | | *MAT***a** *rad52-F186A rad59Δ ade2-1 can1-100 his3-11,15 leu2*Δ*BstEII lys2 TRP1 ura3-1*  *MAT*α *rad52-F186A rad59Δ ade2-1 can1-100 his3-11,15 leu2*Δ*EcoRI LYS2 trp1-1 ura3-1* |

^a^All strains are made in the W303 background except that they are RAD5

^b^{Zou, 1997 7460 /id}

^c^{Lisby, 2003 8429 /id}
